# Clinical and radiographic outcomes following transcrestal maxillary sinus floor elevation with injectable xenogenous bone substitute in gel form: a prospective multicenter study

**DOI:** 10.1186/s40729-022-00431-5

**Published:** 2022-07-22

**Authors:** Teresa Lombardi, Luca Lamazza, Fabio Bernardello, Grzegorz Ziętek, Claudio Stacchi, Giuseppe Troiano

**Affiliations:** 1 Department of Health Sciences, Magna Græcia University, 88100 Catanzaro, Italy; 2 Department of Oral and Maxillofacial Sciences, Sapienza University of Rome, 00161 Rome, Italy; 3 Studio Bernardello, via Bonvicini, 42, Terranegra di Legnago (VR), 37045 Verona, Italy; 4 Ziętek Clinic, 31315 Kraków, Poland; 5 Department of Medical, Surgical and Health Sciences, University of Trieste, 34100 Trieste, Italy; 6 Department of Clinical and Experimental Medicine, University of Foggia, 71122 Foggia, Italy

**Keywords:** Maxillary sinus augmentation, Transcrestal approach, Gel graft

## Abstract

**Purpose:**

To investigate clinical and radiographic outcomes of transcrestal maxillary sinus floor elevation performed with an injectable xenograft in gel form, analyzing general, local and surgical variables possibly influencing the results.

**Methods:**

Patients with residual crestal height < 5 mm underwent transcrestal sinus floor elevation with xenograft in gel form to allow the placement of a single implant. Simultaneous implant placement was performed when primary stability was ≥ 15 Ncm. Graft height was measured immediately after surgery (T0) and after 6 months of healing (T1). Univariate and multivariate regression models were built to assess associations between clinical variables with implant survival and graft height at T1.

**Results:**

71 patients underwent transcrestal sinus floor elevation and 54 implants were simultaneously placed. Delayed implant placement (at T1) was possible in 5 cases out of 17 (29.4%), whereas in 12 patients (70.6%) implant insertion was not possible or required additional sinus grafting. Implant survival rate, with a follow-up varying from 12 to 32 months after loading, was 100%. Mean pre-operative bone height was 3.8 ± 1.0 mm, at T0 was 13.9 ± 2.2 mm and at T1 was 9.9 ± 2.8 mm. Bone height at T1 was negatively influenced by membrane perforation at surgery (*p* = 0.004) and positively influenced by immediate implant insertion (*p* < 0.001).

**Conclusions:**

Transcrestal sinus floor elevation performed with injectable xenograft gel resulted in 100% implant survival rate. However, immediate implant insertion seems a crucial factor to preserve vertical bone gain: one-stage technique seems to be the most predictable approach to optimize clinical outcomes with this approach.

*Trial registration* clinicaltrials.gov, NCT05305521. Registered 31 March 2022—Retrospectively registered, https://clinicaltrials.gov/ct2/show/NCT05305521.

## Background

Adequate alveolar ridge volume and good bone quality are factors of utmost importance to achieve osseointegration and ensure long-term clinical success of dental implants. Regrettably, poor bone quality and insufficient residual bone height are common issues in patients in need of an implant-supported rehabilitation of edentulous posterior maxilla: in this anatomical district, post-extractive bone resorption is associated with maxillary sinus pneumatization, often resulting in insufficient bone height for implant placement [1–5]. Maxillary sinus floor elevation has expanded therapeutic options by increasing available bone height to allow implant placement in atrophic posterior maxilla. The original technique, first published by Boyne and James [6], is based on a surgical access to the antral cavity performed on the lateral sinus wall, followed by sinus membrane elevation and subsequent grafting of the sub-antral space with autologous bone. Many variations of this surgical technique have been described over time, including the osteotome technique, introduced by Summers in 1994 [7], in which a transcrestal approach was proposed with the aim to decrease morbidity and invasiveness of the intervention. Besides the use of osteotomes, other transcrestal approaches are available today: the access through maxillary sinus floor may be also performed by using specially designed burs [8–10] or piezoelectric tips [11]. Once the sub-antral space has been reached, sinus membrane has to be properly detached and elevated, after checking its integrity with visual inspection and Valsalva maneuver. Membrane elevation may be performed by progressive increments of grafting material inserted through the crestal osteotomy or by exploiting the hydrodynamic action of fluids (i.e., saline solution) injected into the sub-antral space [12–16]. Even if graftless procedures have been described [17, 18], the use of bone substitutes in transcrestal approach allows significantly higher vertical bone gain [19, 20] and can be applied also in cases with residual crestal bone height < 5 mm [21–23]. Clinical and histomorphometric studies indicated no superiority of a specific bone substitute on the others, but highlighted that graft conversion into new bone is heavily influenced by sinus anatomy: predictable regenerative results with transcrestal technique can be obtained only in narrow sinus cavities [24–29].

The use of injectable grafts in gel form, namely the gel-pressure technique, has been proposed for transcrestal maxillary sinus floor elevation by Pommer and Watzek in 2009 [30]. These biomaterials are composed by micronized allogeneic, xenogenous or alloplastic graft particles embedded in collagen matrix or in water-based gels, being characterized by pasty consistency and smooth surface which is likely to prevent accidental Schneiderian membrane perforation during elevation. This surgical technique exploits the hydraulic pressure applied during gel graft injection through the crestal antrostomy to detach the sinus membrane from bony walls and simultaneously fill the sub-antral space, with remarkable shortening of surgical time. However, a possible drawback related to the use of injectable biomaterials is their limited volumetric stability during healing period. Areas grafted with biomaterials in gel form demonstrated increased shrinkage when compared to conventional particulate grafts [31]: especially, when two-stage augmentation is performed, the absence of sinus membrane support provided by the implant apex could result in insufficient regenerated tissue volume after healing.

The present prospective study aims to investigate clinical and radiographic results of transcrestal maxillary sinus floor elevation performed with an injectable xenogenous bone substitute in gel form, and to analyze general, local and surgical variables possibly influencing therapy outcomes.

## Methods

### Study protocol

The present multicenter prospective study was reported following STROBE (STrengthening the Reporting of Observational studies in Epidemiology) guidelines [32]. All procedures were performed in full accordance with the recommendations for investigations with human subjects expressed in the Fortaleza revision of the Declaration of Helsinki (2013). The study protocol had been approved by the relevant ethical committee (Comitato Etico Regione Calabria—Sezione Area Centro n. 115/2021) and recorded in a public registry of clinical trials (www.clinicaltrials.gov—NCT05305521). All patients, after being thoroughly informed about the study protocol, the treatment with its alternatives and any potential risk related to the therapy, signed a written informed consent for the participation in the study and authorized the use of their data for research purposes.

### Selection criteria

Any partially edentulous patient needing unilateral sinus floor elevation for the placement of one dental implant supporting a single crown was eligible for entering this study. Patients were consecutively enrolled, provided that they fulfill the following inclusion criteria:

- Residual bone crest height < 5 mm and width ≥ 6 mm in the planned implant site;
- Healed bone crest (at least 6 months elapsed from tooth loss/extraction);
- Age > 18 years;
- Written informed consent given.

Patients were excluded from this study if presenting one or more of the following general exclusion criteria:

- Absolute medical contraindications to implant surgery [33];
- Uncontrolled diabetes (HBA1c > 7.5%);
- Treated or under treatment with antiresorptives;
- Irradiated in the head and neck area in the last 5 years;
- Pregnant or breastfeeding;
- Substance abusers;
- Psychiatric problems or unrealistic expectations;
- Patient not fully able to comply with the study protocol.

Local exclusion criteria consisted of the following:

- Large sinus cavity (distance > 12 mm between buccal and palatal walls at 10-mm level, comprising the residual alveolar crest) [27];
- Maxillary sinus conditions contraindicating sinus floor elevation [34];
- Poor oral hygiene and motivation (Full Mouth Plaque Score > 20% and or Full Mouth Bleeding Score > 10%).

### Pre-surgical phase

Before surgery, patients enrolled in the present study underwent clinical examination, including periodontal chart and periapical radiographs. Periodontal patients underwent causal therapy at first, were then re-evaluated and, if necessary, received further periodontal therapy before being cleared for sinus augmentation. Anatomy (maxillary sinus bucco-palatal width and presence of septa), conditions of the maxillary sinus (patency of ostiomeatal complex, membrane thickness and eventual presence of sinus pathologies) and residual alveolar crest (width and height) were evaluated by cone beam computed tomography (CBCT). All patients received oral hygiene instructions and professional deplaquing one week prior to surgery and were prescribed with chlorhexidine digluconate 0.2% mouthwash twice a day until the day of surgery.

### Surgical procedure

**Fig. 1:**
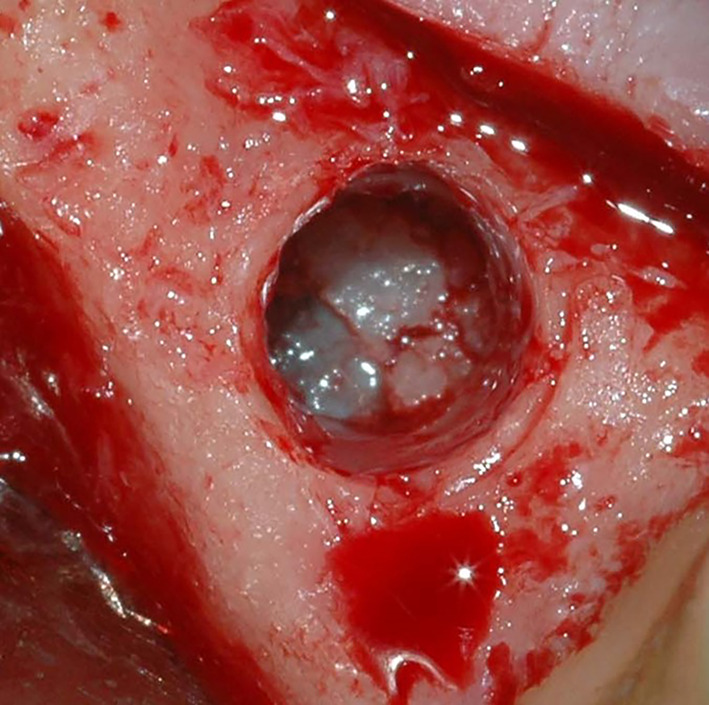
Direct visual check of Schneiderian membrane integrity after crestal osteotomy performed with specific burs for transcrestal sinus approach

**Fig. 2:**
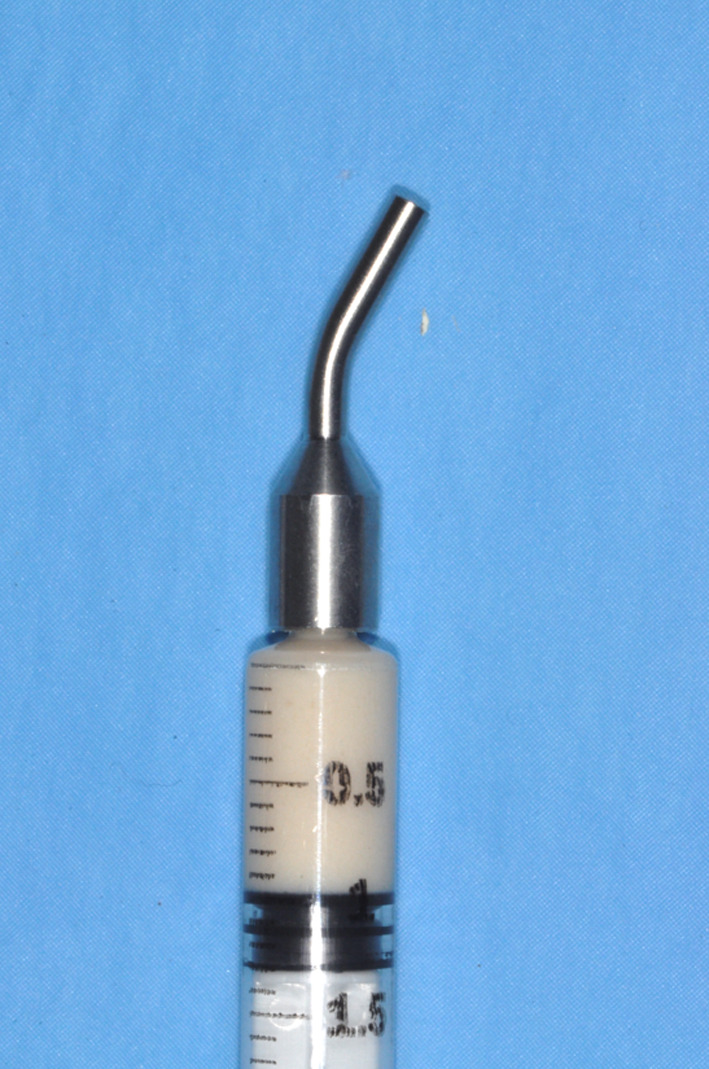
Syringe of xenogenous porcine bone substitute in gel form with stainless steel insert to facilitate graft injection

**Fig. 3:**
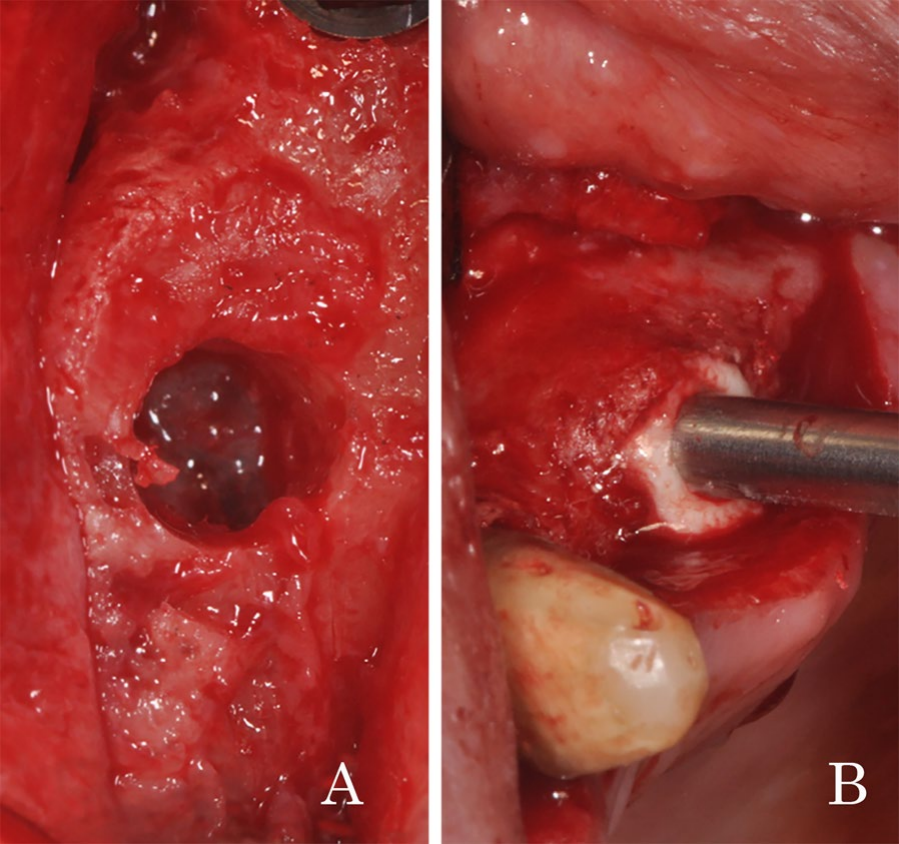
After performing crestal osteotomy and checking the Schneiderian membrane integrity (**A**), the graft is directly injected into the sub-antral space (**B**)

**Fig. 4:**
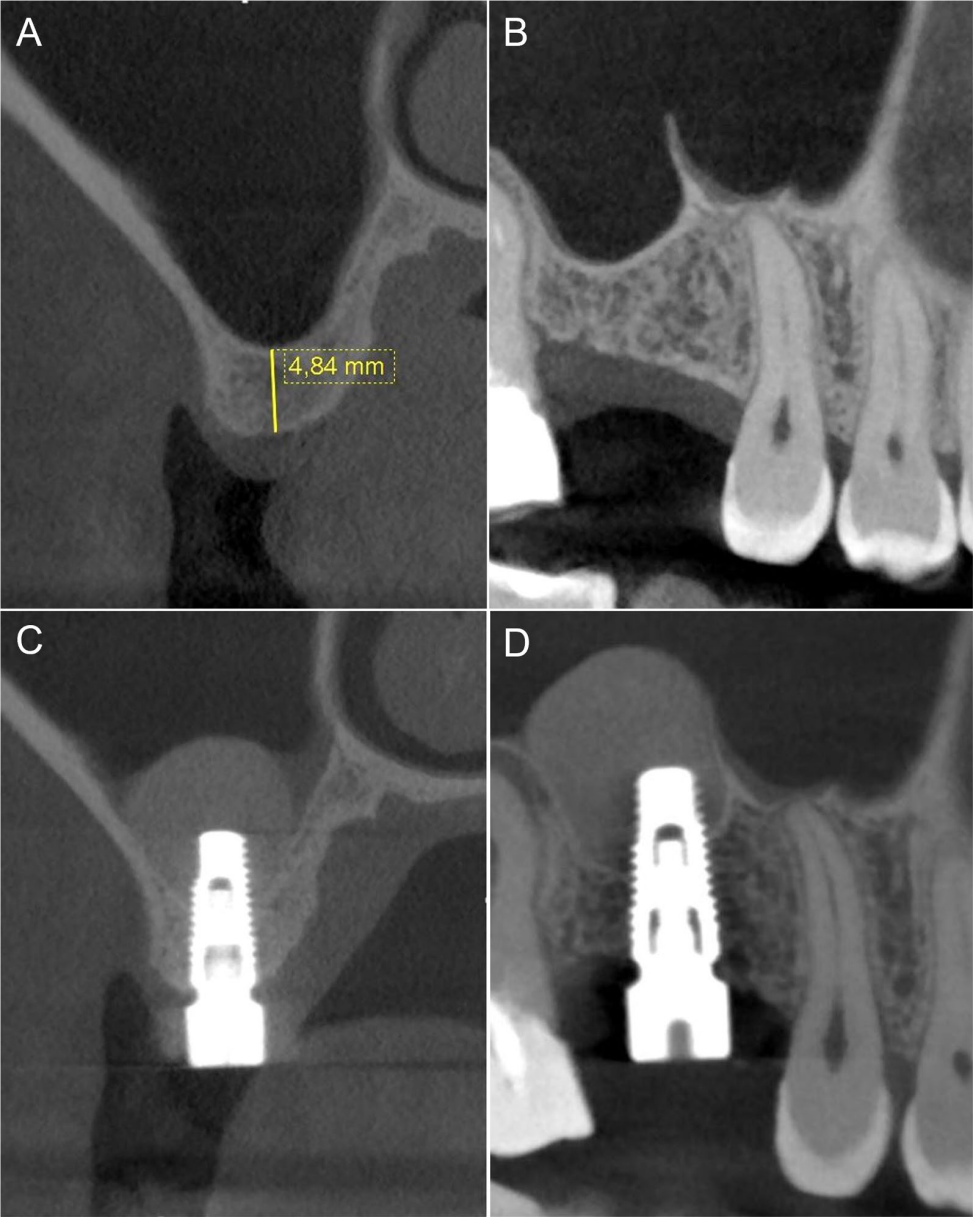
Pre-surgical CBCT with cross-section showing a narrow sinus anatomy and RBH < 5 mm (**A**) and panorex image highlighting the presence of a sharp Underwood septum (**B**). Post-operative CBCT with cross-section showing abundant amount of gel graft apically to implant apex to counteract the expected shrinkage during the healing phase (**C**) and panorex confirming sinus membrane integrity (**D**)

The time taken for each intervention was recorded using a digital chronometer (HS-80TW-1EF, Casio, Tokyo, Japan). The time measurement began with flap incision and ended with the placement of the final suture. Under local anesthesia (articaine 4% with epinephrine 1:100.000—Ubistesin Forte, 3 M ESPE, Seefeld, Germany), a minimally invasive full-thickness flap was elevated to expose the alveolar crest. Clinicians were left free to choose their preferred transcrestal antrostomy technique. After checking sinus membrane integrity, within the visual limitations of this surgical approach, with direct inspection and Valsalva maneuver (Fig. 1), pre-heated (40 °C) xenogenous porcine bone substitute in gel form (Gel 40, Tecnoss, Giaveno, Italy) was injected through the crestal antrostomy in order to elevate the membrane and fill the sub-antral space (Figs. 2, 3). Duration of graft injection (in seconds) was recorded using a digital chronometer. Periapical intra-operative radiographs were taken to confirm membrane elevation of at least 10 mm. Simultaneous implant placement was performed when it was possible to achieve adequate primary stability in the residual crestal bone (peak insertion torque ≥ 15 Ncm), otherwise crestal antrostomy was sealed by collagen sponges (Hemocollagene, Septodont, Saint-Maur-des-Fossés, France) and implant insertion was postponed (Fig. 4).

Flaps were sutured with Sentineri technique [35] and single stitches using synthetic monofilament for a submerged healing of implants. After performing a low-irradiation control CBCT, patients received post-operative instructions specific for sinus surgery (e.g., sneeze with mouth open, avoid nose blowing). Patients underwent antibiotic therapy for 6 days (amoxicillin 1 g three times a day), nonsteroidal anti-inflammatory drugs (ibuprofen 600 mg), when needed, and chlorhexidine digluconate 0.2% mouthwash twice a day for one week. Sutures were removed after 10 days and patients were recalled two times during the first month after surgery and then monthly to check the course of healing. After 6 months, a CBCT scan was performed to evaluate the outcome of the regenerative procedure and to plan implant insertion in cases where placement was not possible at sinus augmentation surgery. All implants (immediately inserted and delayed) were restored after 6 months of submerged healing with screw-retained single metal–ceramic crowns and followed at least for 12 months after prosthetic loading.

### Radiographic measurements

Measurements were taken on CBCTs performed immediately after surgery (T0) and at 6 months (T1) by a calibrated examiner (C.S.), using the specific tool of an imaging software (OsiriX MD, Pixmeo, Bernex, Switzerland).

Distances were measured on the four CBCT cross-sectional slices (step 1 mm; width 1 mm) corresponding to the position where implant insertion was planned and the mean of 4 measurements was considered in the subsequent analysis. The following measures were taken: (1) residual bone height (RBH) between the alveolar crest and the sinus floor; (2) sinus width (SW) (distance in mm between buccal and palatal walls at 10-mm level, comprising the residual alveolar crest); (3) graft height (BG): (distance in mm from the cortical of the sinus floor to the most apical level of the grafted area).

Examiner calibration was performed by measuring BG on a sample of fifteen CBCT cross-sectional slices not included in the study, with a different author (F.B.) serving as reference examiner. Cohen’s k coefficient for intra-examiner and inter-examiner agreement were 95.2% and 92.4%, respectively, for linear measurements within ± 0.1 mm.

### Predictor and outcome variables

This prospective study tested the null hypothesis of no differences in implant survival and vertical bone gain after transcrestal sinus augmentation with injectable gel graft between immediate and delayed implant insertion against the alternative hypothesis of a difference.

The primary predictor variable was the timing of implant insertion.

Primary outcome measure:

- Implant survival after one year of prosthetic loading.
- Secondary outcome measures:
- Vertical bone gain (BG) 6 months after sinus augmentation;
- % graft shrinkage (%GS) during the 6-month healing period;
- Occurrence of any complication or adverse event.

### Statistical analysis

An independent investigator (G.T.) performed data analysis by using the software STATA 16.0 (StataCorp, College Station, USA). Descriptive exploratory analysis of clinical factors was performed by calculating frequencies and percentages for categorical variables, and mean with standard deviation for continuous covariates. In addition, univariate and multivariate non-parametric series regression models were built to assess the association between clinical and radiographic variables with the above-mentioned outcomes. In particular, variables showing significance at the univariate analysis were selected and included in the multivariate model. A *p*-value lower than 0.05 was used as a threshold of statistical significance.

## Results

### Study population and clinical results

Seventy-one consecutive patients (35 females and 36 males; age range between 25 and 83 years, mean 57.5 ± 11.7 years; 21 smokers, 50 no smokers) were included in this study and underwent transcrestal floor elevation. Surgeries were performed between August 2018 and May 2020 by four experienced operators (CS *n* = 22; TL *n* = 11; GZ *n* = 4; FB *n* = 34). No drop-outs were recorded during the entire study period.

Transcrestal osteotomy was performed with osteotomes (*n* = 15; 21.1%), piezoelectric inserts (*n* = 9; 12.7%) or specially designed burs (*n* = 47; 66.2%). Mean surgical time for the entire intervention was 27.2 ± 11.3 min (range 14–54 min), with no significant differences among the three techniques. Mean operative time for graft insertion was 58.1 ± 31.0 s (range 11–176 s). In spite of negative Valsalva maneuver, three small perforations had been highlighted by post-operative radiographs in three patients (4.2%): two perforations occurred when using osteotomes (13.3% of osteotome cases), one perforation with burs (2.1% of bur cases). The small quantity of disseminated gel graft was spontaneously cleared by ciliary activity through ostiomeatal complex and no graft remnants were detectable at CBCT examination after 6 months. No other intra- or post-operative complications or adverse events were recorded in this study. Demographic and surgical characteristics are summarized in Table 1.

**Table 1:**
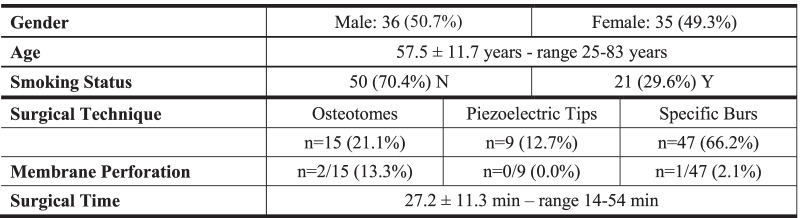
Demographic and surgical characteristics of the included patients

Data are expressed as mean ± standard deviation. *N*: no; *Y*: yes; *n*: number; *min*: minutes

Simultaneous sinus floor elevation and implant placement was performed in 54 cases (76.1%), while in 17 cases (23.9%) implant insertion was not possible due to insufficient primary stability. In five out of these 17 cases (29.4%), implant could be placed after graft consolidation, whereas in 12 patients (70.6%) implant insertion was not possible due to limited available bone height or required additional sinus grafting. All inserted implants (*n* = 59) were satisfactorily in function with a follow-up varying from 12 to 32 months (mean 22.9 ± 5.8 months) after prosthetic loading (100% survival rate).

### Radiographic measurements

Mean pre-operative available bone height was 3.8 ± 1.0 mm (range 1.2–4.9 mm), immediately after surgery was 13.9 ± 2.2 mm (range 10.3–22.8 mm) and after 6 months of healing was 9.9 ± 2.8 mm (range 1.2–13.9 mm). Mean vertical bone gain (BG) 6 months after sinus augmentation was 6.1 ± 2.2 mm (range 0–11.1 mm). Mean BG in one-stage cases (6.7 ± 1.5 mm) resulted significantly higher than BG in cases where two-stage approach was performed (4.1 ± 2.9 mm). Univariate analysis demonstrated a significant negative influence of membrane perforation on BG (*p* = 0.004) and a significant positive effect on BG of immediate implant insertion (*p* < 0.001).

**Fig. 5:**
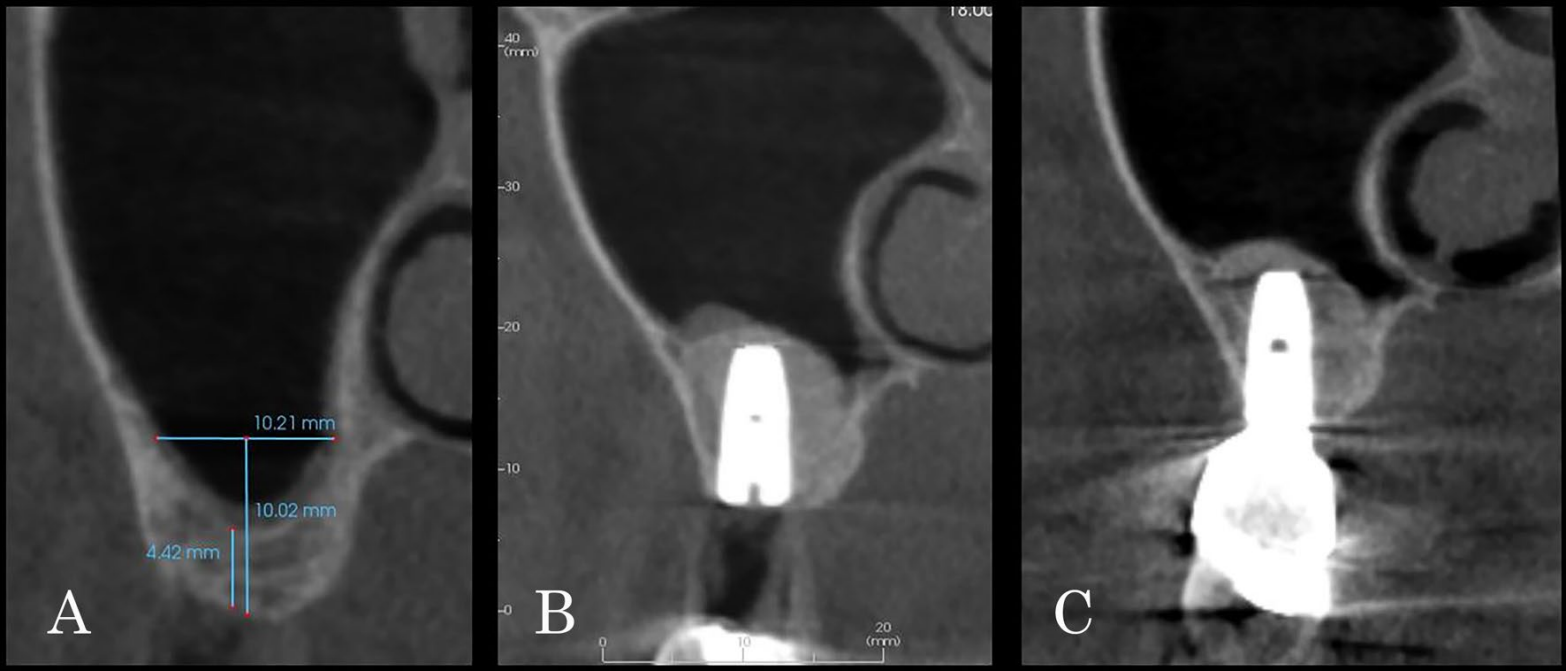
CBCT cross-section images taken presurgically (**A**), at T0 (**B**) and T1 (**C**) showing the “tent-pole” effect of the implant apex

**Fig. 6:**
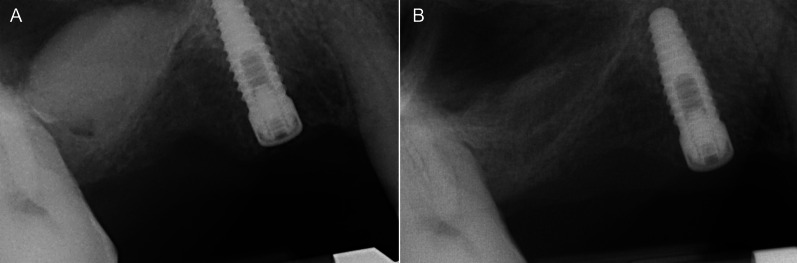
Periapical radiographs taken immediately after transcrestal sinus floor elevation without simultaneous implant insertion (**A**) and after 6 months of healing (**B**): an evident shrinkage of the regenerated volume is present

Mean percentual graft shrinkage (%GS) during the healing period was 36.1 ± 22.9% (range 2.8–100%). Multivariate analysis highlighted a significant inverse correlation between %GS and implant length (*p* = 0.018), significantly increased %GS in presence of membrane perforation (*p* = 0.004) and strong influence of immediate implant insertion in limiting %GS (*p* < 0.001) (Figs. 5, 6).

More details about the association of clinical variables with the analyzed outcomes are reported in Tables 2 and 3.

**Table 2:** Univariate regression model for the outcome “Vertical Bone Gain”

Number of cases = 71	Univariate analysis		
Vertical bone gain	Coeff	[95% CI]	*p*-value
Age	− 0.01	[− 0.07–0.04]	0.662
Gender			
Female	1		
Male	− 0.08	[− 1.12–0.96]	0.882
History of periodontitis			
No	1		
Yes	− 0.35	[− 1.34–0.64]	0.487
Smoking			
No	1		
Yes	0.30	[− 0.72–1.32]	0.558
Residual bone height	0.69	[− 0.45–1.84]	0.236
Sinus width			
Narrow	1		
Wide	− 0.94	[− 2.71–0.84]	0.302
Surgical technique			
Osteotomes	1		
Burs	− 0.40	[− 1.97–1.16]	0.615
Piezoelectric tips	− 2.42	[− 5.15–0.31]	0.083
Implant length	− 524.5	[− 1075.7–26.62]	0.062
Membrane perforation			
Absent	1		
Present	− 3.01	[− 5.06 to − 0.95]	0.004*
Implant insertion			
Immediate	1		
Delayed	− 2.64	[− 4.06 to − 1.22]	0.000*

*Coeff.* coefficient, *CI* confidence interval, * *p*-value < 0.05

**Table 3:** Univariate and multivariate regression models for the outcome “Graft Shrinkage”

Number of cases = 71	Univariate analysis			Multivariate analysis		
Graft shrinkage	Coeff	[95% CI]	*p*-value	Coeff	[95% CI]	*p*-value
Age	− 0.02	[− 0.44–0.40]	0.942			
Gender						
Female	1					
Male	− 2.84	[− 13.56–7.88]	0.604			
History of periodontitis						
Yes	1					
No	− 2.19	[− 12.2–7.82]	0.669			
Smoking						
Yes	1					
No	− 11.98	[− 21.88–− 2.08]	0.018*			
Residual bone height	− 7.88	[− 18.28–2.52]	0.138			
Sinus width						
Narrow	1					
Wide	6.64	[− 12.09–25.37]	0.487			
Surgical technique						
Osteotomes	1					
Burs	− 13.70	[− 26.34–− 1.06]	0.034*			
Piezoelectric tips	11.30	[− 16.62–39.23]	0.428			
Implant length	− 5.33	[− 9.97– to 0.69]	0.024*	− 3.60	[− 6.55–0.65]	0.018*
Membrane perforation						
Absent	1			1		
Present	32.06	[10.43–53.69]	0.004*	28.08	[9.25–46.91]	0.004*
Implant insertion						
Immediate	1			1		
Delayed	36.67	[24.66–48.68]	0.000*	25.77	[17.56–33.98]	0.000*

*Coeff.* coefficient, *CI* confidence interval, *: *p*-value < 0.05

## Discussion

Bone regeneration in the maxillary sinus cavity occurs as a direct biological response to the surgical trauma. Membrane detachment from the bony walls delimits a secluded sub-antral space filled by blood clot, which is subsequently colonized by newly formed vessels and osteoprogenitor cells migrating from the denuded sinus floor and walls [28]. The contribution of the Schneiderian membrane to this process has been widely debated, but current evidence does not consistently support a significant osteogenic role for sinus membrane following maxillary sinus augmentation procedures [36]. Therefore, adequate membrane elevation is a fundamental prerequisite for bone regeneration, favoring neo-angiogenesis and cellular colonization of the newly formed sub-antral space. Previous studies showed a direct correlation between adequate membrane elevation, new bone formation, and volumetric stability of the regenerated tissue [25, 27, 37]. In transcrestal approaches, correct membrane elevation seems to occur predictably only in narrow sinuses [27, 29]: for this reason, large sinus cavities (> 12 mm between buccal and palatal walls at 10-mm level, comprising the residual alveolar crest) were excluded from the present study. Indirect hydrodynamic membrane detachment by gel graft injection resulted fast and effective: mean operative time for membrane elevation and bone grafting was 58.1 ± 31.0 s, in perfect accordance with a previous investigation performed by injecting nanocrystalline hydroxylapatite in aqueous paste (50.0 ± 8.0 s) [38]. Duration of the entire intervention was considerably shortened by the use of injectable biomaterials: mean surgical time recorded in the present study (27.2 ± 11.3 min) is greatly reduced if compared to a recent randomized controlled trial in which, after transcrestal hydrodynamic membrane elevation, progressive increments of a xenograft in granular form were performed (mean duration 48.1 ± 11.1 min) [39].

No implant loss was recorded during the entire follow-up period in the present study. However, in case of two-stage transcrestal augmentation, only 5 implants out of 17 (29.4%) could be placed after 6 months of healing due to graft shrinkage (GS). One of the main functions of osteoconductive bone substitutes in maxillary sinus augmentation is to provide adequate mechanical support to blood clot during the healing phase, opposing the positive pressure of the maxillary sinus, thus facilitating the formation of a sufficient amount of new bone [40, 41]. The findings of the present investigation seem to indicate that the mechanical properties of the collagenated xenogenous gel used in this study (40% of collagen content) are not sufficient to effectively support sinus membrane during the healing period. As a matter of fact, multivariate analysis confirmed that immediate implant insertion is the most crucial factor to reduce %GS and highlighted a significant inverse correlation between %GS and implant length. These data are in accordance with previous studies showing that two-stage sinus floor augmentation performed by using grafts with low volumetric stability failed in obtaining sufficient bone increase for subsequent implant installation. Simultaneous implant insertion exerts a “tenting” effect on the sinus membrane, securing the grafted area against the continuous positive air pressure present in the sinus cavity [42–46].

Mean bone gain (BG) after 6 months of healing was 6.1 ± 2.2 mm, greater than mean BG recorded in previous meta-analyses analyzing studies on transcrestal sinus floor elevation with grafting materials. Antonaya-Mira and co-workers (2012) reported BG ranging from 2.07 to 4.62 mm [20], more recently Yan et al. (2018) found BG values ranging from 3.56 to 5.00 mm [47]. Graftless transcrestal approaches have also been described and studied: a recent meta-analysis demonstrated satisfactory results in terms of implant survival rate with this technique, but limited bone gain (mean 2.4 mm) [48]. The use of grafting materials seems to increase the probability of regenerating a greater amount of new bone, and should be preferred in presence of limited RBH (< 5 mm) [20, 29, 49].

The remarkable BG recorded in the present study could be explained on one side by the hydrodynamic properties of injectable gel, effectively exploiting Pascal law to elevate sinus membrane, and on the other side by the exclusion of large sinus cavities from this investigation. Univariate analysis showed that immediate implant insertion, besides the aforementioned preventive role in reducing GS, had a significant positive effect on BG.

Three membrane perforations, undetected during the surgical procedure, were highlighted by post-operative radiographs. The incidence of this complication (4.3%) is in line with data reported in literature for transcrestal sinus floor elevation [50, 51], and our findings also confirmed the difficulty of a proper intra-operative diagnosis of membrane perforation with this blind surgical technique. The small quantity of accidentally dispersed gel graft was completely cleared through ostiomeatal complex during the healing period, without any sign or symptom related to possible sinus disease. This clinical observation confirms the findings of a recent study, suggesting that bone substitutes in gel form could represent an interesting alternative to granular grafts for their easier clearance from maxillary sinus cavity in case of accidental dissemination, due to the very small dimension of the graft particles (0.3 mm) [52]. Unsurprisingly, multivariate analysis demonstrated significantly increased %GS in presence of membrane perforation and a significant negative influence of this intra-operative complication on BG.

Main limitations of the present investigation are the unbalanced distribution of patients between one-stage and two-stage technique, the exclusion of large sinus cavities from the study and the lack of histological analysis of the regenerated tissue. In addition, as a single injectable bone substitute in gel form was used, the present results should not be automatically generalized to other biomaterials of the same category.

## Conclusions

Transcrestal sinus floor elevation performed in crests with reduced bone height (< 5 mm) by using injectable xenogenous bone substitute in gel form resulted in 100% implant survival with a follow-up varying from 12 to 32 months after prosthetic loading. However, the results of the present prospective clinical study suggest that immediate implant insertion is a crucial factor to preserve vertical bone gain and reduce graft shrinkage: one-stage technique seems to be the most predictable approach to optimize clinical outcome with this surgical procedure.

## Data Availability

The datasets used and analyzed during the current study are available from the corresponding author on reasonable request.

## Abbreviations

BG: Bone gain
CBCT: Cone beam computed tomography
GS: Graft shrinkage
RBH: Residual bone height
SW: Sinus width
